# Factors associated with parental intention to vaccinate their child against influenza, Finland, February to March, 2022: a web-based survey

**DOI:** 10.2807/1560-7917.ES.2023.28.49.2200828

**Published:** 2023-12-07

**Authors:** Idil Hussein, Simopekka Vänskä, Jonas Sivelä, Tuija Leino, Hanna Nohynek

**Affiliations:** 1 Department of Health Security, Finnish Institute for Health and Welfare (THL), Helsinki, Finland; 2 Doctoral Programme in Population Health, Faculty of Medicine, University of Helsinki, Finland

**Keywords:** influenza, vaccine uptake, national vaccination programme, public health, vaccination intention

## Abstract

**Background:**

Influenza vaccination for children aged 6 months to 6 years is included in the national vaccination programme in Finland. Although all vaccines in the programme are free of charge, national coverage of influenza vaccination among children under 3 years and 3–6 years during 2020/21 was 43% and 35% respectively, with regional differences.

**Aim:**

To assess factors underlying parental vaccination intention in order to increase influenza vaccine uptake among children.

**Methods:**

We conducted a web-based survey among parents (n = 17,844) of randomly selected eligible children (aged 6 months–6 years) in February–March 2022 in five Finnish municipalities from regions of high and low coverage. Logistic regressions were used to determine associations between vaccination intention and e.g. sociodemographic factors, attitudes and knowledge. Linkage to the national vaccination register was used to confirm realisation of vaccination intention after the study.

**Results:**

Participation rate was 13% (n = 2,322 parents). Influenza knowledge, trust in official information, responding parent’s education level, adherence to the vaccination programme, number of children and changes in attitudes towards vaccination since COVID-19 were all associated with intention to vaccinate. Vaccination intention for children was 64%, and realised vaccination 51%.

**Conclusion:**

Despite the low participation rate, both vaccinated and unvaccinated children were represented. Influenza vaccine uptake is not dependent on a single factor. Our results identified the need for open dialogue between parents and healthcare professionals, as the lack of vaccine being offered by healthcare professionals was the most reported reason for not vaccinating.

**Key public health message**

**What did you want to address in this study?**

In Finland, childhood vaccines have generally a high uptake of well over 90% for most vaccines. However, influenza vaccine has a suboptimal coverage, with regional differences in uptake. We wanted to address factors affecting influenza vaccine uptake through a survey to parents with young children (≤ 6 years). Furthermore, we wanted to find out whether the children of parents who completed the survey actually did receive their influenza vaccine.

**What have we learnt from this study?**

Our results show that parental vaccine decision-making is not always driven by intention to vaccinate. Parents who indicated no intention to vaccinate maintained this standpoint, and nearly one fourth of children whose parent had a positive intention did not receive their vaccine. The most commonly reported reason for not vaccinating one’s child was that the vaccine had not been offered at the health clinic.

**What are the implications of your findings for public health?**

Our findings increase the understanding of factors associated with regional influenza vaccine uptake in Finland. Moreover, our findings could help decisionmakers understand the complexity of vaccination uptake, and also serve as a reminder for healthcare professionals to proactively engage in open dialogue with parents regarding the influenza vaccine.

## Introduction

Vaccinations have had an important contribution to global health, as they greatly reduce disease, disability and inequity [1]. However, parental concerns, among other factors, can lead to delaying or even refusing vaccination, thereby decreasing overall vaccine coverage [1]. Influenza is an infectious disease affecting the upper and lower respiratory tract, with annual outbreaks being the highest in children [2]. Approximately 870,000 children under 5 years of age worldwide are hospitalised annually because of influenza, and it is estimated that between 28,000 and 111,500 deaths in this age group can be attributed to influenza each year [3]. Certain vulnerable groups are at an increased risk of developing severe illness, complications and mortality [3,4]. The burden of influenza is high, particularly in children under 5 years, and hence the World Health Organization recommends vaccinating all children between the ages of 6 months to 5 years [5]. Influenza vaccination is an effective way to protect children from influenza, and its secondary diseases, such as acute otitis media, bronchitis and pneumonia [6]. Yet, only a few European countries, including Austria and the United Kingdom among others, implement a routine seasonal influenza vaccination for children [7,8].

Influenza vaccine has been a part of Finland’s national vaccination programme (NVP) for adult risk groups since the 1980s [9]. The vaccination programme was expanded to include all children between the ages of 6 and 36 months in 2007, and then to all children between the ages of 3 and 6 years in 2018 [9]. However, the uptake is suboptimal, with only 42.9% of children under the age of 3 years and 34.7% of children aged 3–6 years vaccinated against influenza during the 2020/21 season [10]. Vaccine uptake of other childhood vaccines is significantly higher. For example, the national coverage of the measles-mumps-rubella (MMR) vaccine (first dose) in children born in 2021 was 93.4% [11]. Moreover, refusing childhood vaccines is rare in Finland; only ca 1% of children under the age of 3 years are completely unvaccinated [12].

Disparities between influenza vaccine uptake exist across different age groups and across geographical regions in Finland, and coverage is notably higher in eastern Finland compared with western Finland [10]. Regional differences can be caused by both practical and ideological factors [6], and the information sources parents use can affect the decision to vaccinate their children [13]. Furthermore, it has been hypothesised that parents are more hesitant towards influenza vaccine compared with other routine childhood vaccinations [14]. Public health measures implemented during the COVID-19 pandemic led to a sharp decline in influenza incidence during the first 2 years of the pandemic [15]. However, as we encountered the first influenza epidemic since COVID-19 in 2021, there was an increasing need to understand parental willingness to vaccinate children against influenza.

The aim of this study was to examine factors associated with parents’ intention to vaccinate their child during the 2021/22 influenza season in Finland. For verification, the influenza vaccine uptake among the children of study participants was extracted from the national vaccination registry after a 9-month follow-up timeframe.

## Methods

### Study setting and population

We conducted a quantitative web-based survey by randomly sampling eligible children and their parents from the Finnish Population Information System (PIS). The PIS is a computerised national register that contains basic information about Finnish citizens and foreign citizens residing in Finland on a permanent or temporary basis. The sampled children and their parents lived in four study areas and the children were between the ages of 6 months and 6 years. The study areas were selected based on both their respective vaccination behaviour (high vs. low coverage) and their size [10]. 

Finland consists of 309 municipalities [16], and the four study areas were comprised of five Finnish municipalities (population data source [17]). Espoo (south, 305,274 inhabitants) and Kuopio (east, 122,594 inhabitants) represented traditionally high vaccine coverage areas, and Oulu (north-west, 211,848 inhabitants), and Mustasaari/Pietarsaari (west, 38,896 inhabitants altogether) were low vaccine coverage areas. 

**Figure 1:**
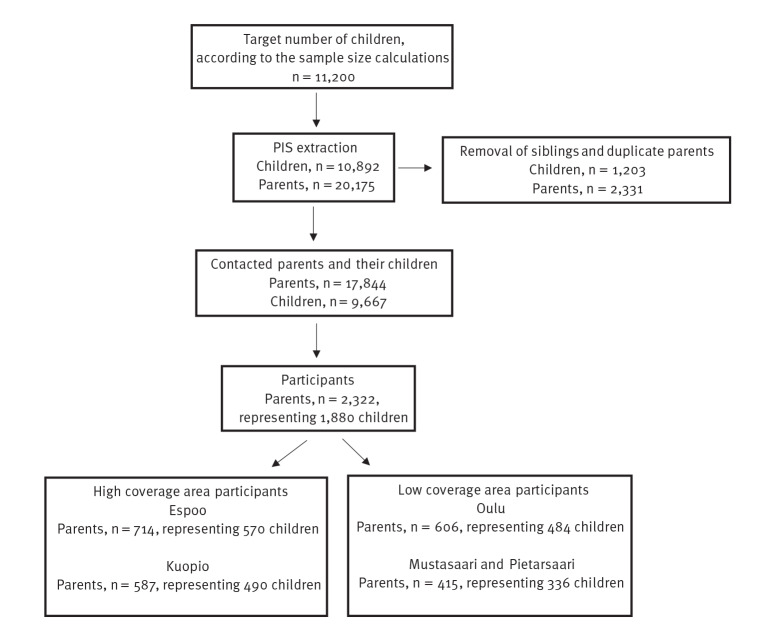
Flowchart of participant selection from the four study areas, municipalities of Espoo, Kuopio, Oulu and Mustasaari/Pietarsaari, Finland, December 2021–March 2022

Figure 1 shows the flowchart of participant selection from the study areas. To achieve representativeness, we aimed to extract randomised data from the PIS on 1,400 eligible children per sex (200 children per age group per study area, at most one child per family; seven age groups: 6 months, 1 year, 2 years, 3 years, 4 years, 5 years and 6 years, based on the age of the child at the time of data extraction), resulting in 11,200 children. Because of the smaller size of some study areas, data on a total of 10,892 children were extracted. For those children, we also extracted the name, sex, year of birth, mother tongue and address of their legal guardian(s) (i.e. parent(s)) from the PIS in December 2021. 

PIS: population information system.

The inclusion criteria for children were two-fold: living in one of the chosen study areas, and being age-appropriate. As we did not want to overburden families by selecting multiple age-appropriate siblings per family and hence having parents contributing more than once, we decided to remove sibling children. In the case of having sampled multiple siblings per family, we included the younger sibling while excluding the older sibling(s) from the study. We were able to include 17,844 parents (representing 9,667 children), and 8,177 children had two legal guardians (i.e. parents).

### Data collection

The data were collected via a quantitative online survey. The survey was created by combining questions used in the Eurobarometer [18], which is a tool measuring the public opinion in the European Union since 1974, and the Finnish Science Barometer [19], which is a study conducted every 3 years to investigate the attitudes that people living in Finland have towards science. In addition, questions regarding knowledge and perceptions about influenza and vaccination against the disease were included. The survey was bilingual, using the two official languages of Finland, Finnish and Swedish. The survey was piloted first with a small sample of parents (< 10), who were not included in this study, to ensure the understandability and user-friendliness of the questionnaire. The full survey in English is provided in the Supplement.

All eligible parents were contacted by post in late January 2022 and invited to participate. The first round of contact included the information letter, explaining the credentials (i.e. website, username and password) required to participate in the study, the aim and objectives of the study and information on the rights of the participant. If the parent had not used their personal credentials and logged on to the questionnaire by the end of the second week of February, the participant was contacted by post for a second time with a short letter, reminding them of the study, and kindly requesting them to take part. The survey results were collected between 31 January and 14 March 2022.

Each questionnaire could be answered only once. Parents had the choice of taking a break from answering, and completing the questionnaire later, within the timeframe of the data collection period, i.e. before 14 March 2022. A large majority of children (76.5%, 1,438/1,880) had one parent participating in the study. For the remaining 23.5% (442/1,880) of children, they had two participating parents. No separate consent form was used, as parents consented by completing the survey. 

### Survey contents

The web-based survey consisted of 28 questions divided under five topics: information sources and trust, expertise, vaccination behaviour, knowledge of influenza and background information, i.e. education and profession. The first topic, information sources and trust, included two multiple choice questions. The topic of expertise included one multiple choice question and an open-ended question. The vaccine behaviour topic included questions about influenza and other childhood vaccines in the NVP. Parents were asked whether their child has received or will receive the influenza vaccine during the influenza season 2021/22, and they were subsequently asked to rank the influence that different individuals (such as friends, family and healthcare professionals) have had on their vaccination decision. To study the reasons for non-vaccination, the parents who had not vaccinated or intended not to vaccinate their child were asked to expand on the reason(s) for not vaccinating. Parents’ influenza knowledge was measured by using five questions. Knowledge was calculated by assigning one point to each correct answer (including one multiple choice question with each correct answer warranting one point) and then summing up all five questions (maximum score: 11 points). Knowledge levels were then ranked into three categories: excellent (11 points), good (8–10 points) and poor (≤ 7 points) knowledge. 

The vaccination status of the children during the 2021/22 influenza season (1 October 2021–30 June 2022) was verified by linking the child’s unique social security number with the National Vaccination Register, maintained by the Finnish Institute for Health and Welfare (THL). By extending the examined vaccination period to the end of June, we aimed to catch potential late registrations of vaccinations or delays in data transmission. The verification linkage was done on 9 May 2023. 

### Statistical analysis

Univariable and multivariable logistic regression were performed to assess the relationships between the intention to vaccinate and various factors such as sociodemographic variables, trust in information sources and vaccination behaviour. Associations were expressed as odds ratios (OR), and a p value of < 0.05 was considered statistically significant. Variables which were statistically significant in the univariable model were added to the multivariable model. All analysis were performed using SPSS for Windows, version 28 (IBM Corp.).

## Results

### Sociodemographic characteristics and intention to vaccinate

At the end of data collection in March 2022, 2,322 (13%) parents had participated in the survey, representing 1,880 (19.4%) children. The mean age of the children was 2.97 ± 1.99 years, and 50.9% (956/1,880) of the children were male and 49.1% (924/1,880) were female. Table 1 describes the sociodemographic characteristics of the responding parents. Of the responding parents, 38.8% (n = 900) were males, 61.2% (n = 1,422) were females and 77.5% (n = 1,755) had a university-level degree. The majority of parents (90.7%, n = 2,106) spoke one of the two official languages, Finnish or Swedish, as mother tongue, with 7.2% (n = 168) of parents having another mother tongue. Information on mother tongue was missing for 2.1% (n = 48). 

**Table 1:** Sociodemographic characteristics of parents completing the survey, Finland, February–March 2022 (n = 2,322)

Characteristics	n	%
**Sex **		
Male	900	38.8
Female	1,422	61.2
**Age (years)**		
20–24	35	1.5
25–29	224	9.6
30–34	696	30.0
35–39	771	33.2
40–44	448	19.3
45–49	125	5.4
50–54	19	0.8
55–59	3	0.1
≥ 60	1	0.0
Mean ± SD, range		
Responding parent	35.1 ± 5.2	20–51
Other parent^a^	37.4 ± 5.6	23–65
**Responding parent’s level of education **		
Primary school	36	1.6
Vocational school	315	13.6
High school	155	6.7
Bachelor’s degree	726	31.3
Master’s degree	929	40.0
Doctoral degree	144	6.2
Missing	17	0.7
**Other parent’s level of education **		
Primary school	51	2.2
Vocational school	485	20.9
High school	149	6.4
Bachelor’s degree	628	27.0
Master’s degree	826	35.6
Doctoral degree	132	5.7
Missing	51	2.2
**Occupation**^b^		
Executive position	129	5.6
Managerial employee/clerk	854	36.8
Employee	882	38.0
Entrepreneur	114	4.9
Farmer	3	0.1
Student	99	4.3
Stay-at-home parent	138	5.9
Unemployed	29	1.2
Other	47	2.0
Missing	27	1.2
**Study area**		
Espoo	714	30.7
Oulu	606	26.1
Kuopio	587	25.3
Mustasaari/Pietarsaari	415	17.9
**Mother tongue**		
Finnish	1,759	75.8
Swedish	347	14.9
Other	168	7.2
Missing	48	2.1
**Number of children**		
1	702	30.2
2	1,063	45.8
3	368	15.8
4 or more	173	7.5
Missing	16	0.7
**Parents’ relationship**		
Marriage or cohabitation	2,183	94.0
Some other form	123	5.3
Missing	16	0.7
**Family’s combined annual income (EUR)**		
Under 10,000	25	1.1
10–20,000	73	3.1
20–40,000	238	10.3
40–60,000	454	19.6
60–80,000	542	23.4
80–100,000	374	16.1
Over 100,000	466	20.1
Do not want to answer	140	6.0
Missing	10	0.4

SD: standard deviation.

^a^ We collected information on the legal guardians of the child, which were in most cases the parents. The ‘responding parent’ could either be a mother or a father. The ‘other parent’ is the father in most pairs (61.2%); however, there were some pairs where both parents were females or males (estimated to be less than 5 pairs in total).

^b^ Occupational groups were classified according to the classification used by Statistics Finland [24].

The survey questions were not mandatory to answer; hence, some data are missing. Of the responding parents, 89.3% (2,065/2,313) indicated their child has received all offered vaccines in the NVP, while 9.7% (225/2,313) of parents replied they had vaccinated their child with some of the offered vaccines; 0.8% (18/2,313) and 0.2% (5/2,313) of parents indicated they have not given their child any vaccines or do not remember giving their child any vaccines, respectively. When asked about intention, 65.8% (1,525/2,319) of parents indicated intent to vaccinate their child or answered that they had already done so. A total of 29.8% (691/2,319) parents replied they will not be vaccinating their child for influenza this influenza season and 4.4% (103/2,319) of parents were unsure about their child receiving the influenza vaccine this season.

**Figure 2:**
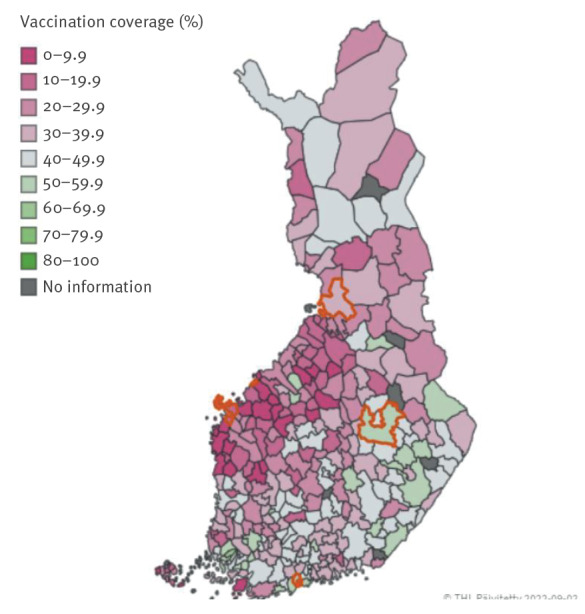
Influenza vaccination uptake for children aged 6–35 months, Finland, 2021/22 season

Figure 2 shows the municipal-level vaccine uptake in Finland during the season of 2021/22 for children aged 6 to 35 months. In total, 34.2% (794/2,319) parents indicated no intention to vaccinate or uncertainty regarding vaccinating their child against influenza, of whom 34.5% (274/794) were from the Mustasaari/Pietarsaari area (low coverage). Among the parents who indicated they did not intend to vaccinate their child against influenza or were unsure, the most reported reasons were that the vaccine was not offered (32.9%, 261/794) and influenza being a harmless disease (27.1%, 215/794). Participants were also asked to indicate which groups of people were entitled to a free-of-charge influenza vaccine in their opinion. Among participants, 74.7% (1,734/2,322) were aware that children between the ages of 6 months and 6 years could be immunised against influenza under the NVP, while 94.6% (2,196/2,322) of the participants knew that those belonging to a risk group are entitled to an influenza vaccine.

Municipal-level influenza vaccination coverage was extracted from THL’s vaccination coverage map [10]. The municipalities chosen for the study are outlined with red: Espoo and Kuopio (high coverage), Oulu and Mustasaari/Pietarsaari (low coverage).

### Factors associated with vaccination intention

Table 2 summarises parents’ responses to survey questions on factors associated with vaccination intention, including religion, information sources and attitudes about vaccines. 

**Table 2:** Survey responses from participating parents on religious practice, information sources and trust, and vaccination behaviour and knowledge , Finland, 2021/22 season (n = 2,322^a^)

Survey topics	n^a^	%
**Religious practice**		
**Actively practice religion**	**2,304**	**99.2**
No	1,921	82.7
Yes	283	12.2
Do not want to tell	100	4.3
**Information sources and trust**		
**Trust in official information^b^**	**2,310**	**99.5**
Not at all or not much	64	2.8
Very much or rather much	2,228	96.0
Do not know	18	0.8
**Trust in journalistic media**	**2,300**	**99.1**
Not at all or not much	650	28
Very much or rather much	1,442	62.1
Do not know	208	9.0
**Trust in social media**	**2,305**	**99.3**
Not at all or not much	2,043	88.0
Very much or rather much	126	5.4
Do not know	136	5.9
**Trust in influencers**	**2,294**	**98.8**
Not at all or not much	2,031	87.5
Very much or rather much	109	4.7
Do not know	154	6.6
**Trust in personal contacts**	**2,297**	**98.9**
Not at all or not much	803	34.6
Very much or rather much	1,127	48.5
Do not know	367	15.8
**Trust in other sources**	**648**	**27.9**
Not at all or not much	173	7.5
Very much or rather much	171	7.4
Do not know	304	13.1
**Vaccination behaviour and knowledge**		
**Adherence to the national vaccination programme**	**2,313**	**99.6**
Yes, all recommended	2,065	88.9
Yes, some of the recommended	225	9.7
No vaccines at all or does not know	23	1.0
**Influenza knowledge**	**2,304**	**99.2**
Poor knowledge	444	19.1
Good knowledge	1,163	50.1
Excellent knowledge	697	30.3
**Change in attitudes since COVID-19**	**2,310**	**99.5**
No, attitude has not changed	1,756	75.6
Yes, attitude is more trusting	215	9.3
Yes, attitude is more cautious	339	14.6

^a^ The n numbers vary in the table because the questions were not compulsory in the questionnaire; parents were able to skip questions.

Table 3 presents the logistic regression analysis of factors associated with parents’ intention to vaccinate their child against influenza. Municipality of residence was statistically significant (p < 0.001) in the multivariable analysis. Parents living in Espoo and Kuopio were 60% more likely to vaccinate their child against influenza compared with parents living in Oulu (OR: 1.59, 95% CI: 1.19–2.13 and OR: 1.54, 95% CI: 1.15–2.13, respectively). Parents in Mustasaari/Pietarsaari were 70% less likely to vaccinate their child against influenza compared with parents living in Oulu (OR: 0.31, 95% CI: 0.20–0.48).

**Table 3:** Logistic regression analysis of factors associated with the intention to vaccinate, Finland, February–March 2022 (n = 2,322 parents)

Variable		Univariable analysis			Multivariable analysis		
		OR	95% CI	p value	OR	95% CI	p value
Municipality of residence	Oulu	Ref.		< 0.001	Ref.		< 0.001
	Espoo	1.53	1.21–1.95	< 0.001	1.59	1.19–2.13	0.002
	Kuopio	1.61	1.25–1.95	< 0.001	1.54	1.15–2.13	0.003
	Mustasaari and Pietarsaari	0.26	0.20–0.34	< 0.001	0.31	0.20–0.48	< 0.001
Age		1.01	0.99–1.02	0.491	NA		
Sex	Male	Ref.		0.429	NA		
	Female	0.93	0.78–1.11				
Mother tongue	Finnish	Ref.		< 0.001	Ref.		0.536
	Swedish	0.25	0.19–0.31	< 0.001	0.81	0.52–1.24	0.331
	Other	0.85	0.61–1.19	0.356	1.10	0.70–1.70	0.676
Actively practice religion	No	Ref.		< 0.001	Ref.		0.416
	Yes	0.72	0.56–0.93	0.011	1.10	0.78-1.53	0.621
	Do not want to tell	0.63	0.42–0.95	0.027	1.44	0.82-2.54	0.203
Responding parent’s educational level^a^	Vocational education	Ref.		< 0.001	Ref.		< 0.001
	Non-vocational education	1.01	0.70–1.43	0.987	0.46	0.29–0.73	< 0.001
	University-level education	2.06	1.62–2.62	<0.001	1.04	0.75–1.47	0.776
Other parent’s educational level^a^	Vocational education	Ref.		< 0.001	Ref.		0.191
	Non-vocational education	1.72	1.22–2.43	0.002	1.40	0.91–2.15	0.122
	University-level education	1.88	1.53–2.31	< 0.001	1.24	0.94–1.63	0.121
Number of children	1	Ref.		< 0.001	Ref.		0.002
	2	1.27	1.03–1.55	0.022	1.15	1.10–2.51	0.015
	3	0.76	0.59–0.99	0.040	1.90	1.27–2.84	0.002
	≥ 4	0.72	0.51–1.01	0.055	1.22	0.78–1.90	0.381
Trust in official information^b^	Not at all or not much	Ref.		< 0.001	Ref.		0.008
	Very much or rather much	8.12	4.39–15.02	< 0.001	3.08	1.42–6.67	0.004
	Does not know	1.51	0.45–5.00	0.501	1.19	0.27–5.19	0.819
Trust in journalistic media	Not at all or not much	Ref.		< 0.001	Ref.		0.689
	Very much or rather much	1.45	1.20–1.76	< 0.001	1.10	0.86–1.41	0.460
	Does not know	0.96	0.70–1.33	0.828	0.98	0.65–1.47	0.919
Trust in social media	Not at all or not much	Ref.		0.099	NA		
	Very much or rather much	0.72	0.50–1.03	0.074			
	Does not know	0.79	0.55–1.13	0.191			
Trust in influencers	Not at all or not much	Ref.		0.003	Ref.		0.2780
	Very much or rather much	0.56	0.38–0.83	0.003	1.62	0.90–2.95	0.110
	Does not know	0.71	0.51–0.996	0.047	1.02	0.66–1.56	0.943
Trust in personal contacts	Not at all or not much	Ref.		0.009	Ref.		0.146
	Very much or rather much	0.75	0.62–0.90	0.003	0.79	0.62–1.01	0.057
	Does not know	0.88	0.67–1.14	0.325	0.81	0.59–1.11	0.194
Trust in other sources	Not at all or not much	Ref.		0.128	NA		
	Very much or rather much	1.42	0.92–2.19	0.116			
	Does not know	1.46	0.99–2.14	0.054			
Adherence to the national vaccination programme	Yes, all recommended	Ref.		< 0.001	Ref.		< 0.001
	Yes, some of the recommended	0.14	0.10–0.19	< 0.001	0.20	0.14–0.29	< 0.001
	No vaccines at all or does not know	0.02	0.003–0.14	< 0.001	0.02	0.003–0.20	< 0.001
Influenza knowledge	Poor knowledge	Ref.		< 0.001	Ref.		< 0.001
	Good knowledge	3.03	2.42–3.80	< 0.001	2.73	2.73–3.60	< 0.001
	Excellent knowledge	5.49	4.22–7.14	< 0.001	4.08	2.96–5.60	< 0.001
Change in attitudes since COVID-19	No, attitude has not changed	Ref.		< 0.001	Ref.		0.039
	Yes, attitude is more trusting	1.00	0.74–1.35	0.995	1.08	0.75–1.58	0.672
	Yes, attitude is more cautious	0.49	0.39–0.62	< 0.001	0.68	0.49–0.93	0.015
Parents’ relationship	Marriage or cohabitation	Ref.		0.013	Ref.		0.073
	Some other form	0.63	0.44–0.91	0.013	0.63	0.38–1.04	0.073

CI: confidence interval; NA: not applicable; OR: odds ratio; Ref.: reference.

^a^ Education categories are defined as follows: vocational school, non-vocational school (i.e. high school) and university-level (i.e. Bachelor’s, Master’s, Doctoral degree).

^b^ Official information is classified as information produced or provided by the Ministry of Social Affairs and Health, the Finnish Institute for Health and Welfare and maternal and child healthcare clinics.

Level of education was significantly associated with intention to vaccinate, but only in terms of responding parents’ education. When comparing to responding parents with a vocational education, those with non-vocational education had 55% lower odds of vaccinating their child against influenza (OR: 0.45, 95% CI: 0.29–0.71). The number of children in the family was also associated with the intention to vaccinate. Parents with two or three children were more likely to vaccinate their child against influenza compared to having one child (OR: 1.66, 95% CI: 1.10–2.51 and OR: 1.90, 95% CI: 1.27–2.84, respectively). However, there was no association between the intention to vaccinate and having four or more children (OR: 1.22, 95% CI: 0.78-1.90).

Participants were asked to indicate their level of trust towards various information sources, and they were given a few examples for each option. Only trust in official information sources (i.e. the Ministry of Social Affairs and Health and the Finnish Institute for Health and Welfare) was significantly associated with vaccination intention in the multivariable analysis (p < 0.001). Parents who indicated they trust official information ‘Very much' or ‘Rather much’ were three times more likely to vaccinate their child against influenza (OR: 3.08, 95% CI: 1.42–6.67), when comparing to parents who indicated no trust at all or very little trust. No significant association was found between trusting other sources of information, e.g. social media or personal contacts, and the intention to vaccinate.

High influenza knowledge was positively associated with the intention to vaccinate. Our analyses show that 30.3% (697/2,304) of parents knew all the answers to the 11 knowledge questions, and 50.5% (1,163/2,304) had good influenza knowledge. Poor knowledge was recorded for 19.3% (444/2,304) of participants. In the multivariable analysis, parents with good knowledge were over twice as likely to vaccinate their child against influenza (OR: 2.73, 95% CI: 2.07–3.60). Parents with an excellent level of knowledge were four times more likely to vaccinate their child (OR: 4.10, 95% CI: 3.00–5.66). Moreover, adherence to the NVP was significantly associated with the intention to vaccinate one’s child. Compared with parents whose children have not missed a single recommended childhood vaccination, parents who have had their children vaccinated with some of the recommended childhood vaccinations were 80% less likely to vaccinate their child against influenza (OR: 0.20, 95% CI: 0.14–0.29). Furthermore, parents who have not given their child any vaccines were even less likely to vaccinate their child against influenza compared with parents whose children received all childhood vaccinations (OR: 0.02, 95% CI: 0.003–0.19).

Mother tongue was not a statistically significant factor in the multivariable analysis. Similarly, no association was observed between religious activity and the intention to vaccinate in the multivariable analysis. In our study, we asked parents whether their attitude towards childhood vaccines has changed during the pandemic. Parents who indicated that their attitude was more cautious towards childhood vaccines were less likely to vaccinate their child compared with parents whose attitude had not changed (OR: 0.68, 95% CI: 0.49–0.93).

We conducted a separate logistic regression analysis of each of the four study areas to examine the factors associated with vaccination intention within the study areas. The association between influenza vaccination intention and other factors of interest varied within each study area when performing univariable and multivariable logistic regressions. 

Table 4 shows the breakdown of parental vaccination intention and the realised vaccination during the influenza season from 1 October 2021 to 30 June 2022, nearly 3 months after the survey was completed. As 23.5% (442/1,880) of children had two participating parents, we selected the answer of the first participating parent to analyse the realisation of parental intention. Among these parents, 64.0% (1,203/1,879) had the intention to vaccinate their child. We found that 76.8% (924/1,203) of children whose parent had intended for their child to receive the influenza vaccination, were vaccinated in real life. Of children whose parent had no intention to vaccinate their child, 3.0% (18/596) did receive the vaccine. The realised vaccination uptake (50.8%) is higher than the national coverage of 36.3% among children 6 months to 6 years during the influenza season of 2021/22 [10].

**Table 4:** Realisation of influenza vaccination according to the national vaccination register on March 2023, according to parental intention, Finland, October 2021–June 2022 (n = 1,879^a^ children)

Variables	Total	Yes^b^	No	Unsure
Children classified by parental vaccination intention^c ^(n)	1,879	1,203	596	80
Children vaccinated by 30 June 2022 according to the register (n)	956	924	18	13
Per cent vaccinated	50.8%	76.8%	3.0%	16.3%

^a^ Of 1,880 children examined in the study, one child was not included in the first variable because the parent did not provide an answer to this particular question in the survey.

^b^ Parents who had already vaccinated their child against influenza and parents who were intending to vaccinate their child against influenza are represented here.

^c^ For children with two responding parents, the answer of the first parent was used.

## Discussion

In this study conducted in Finland, we describe numerous factors that are associated with parental intention to vaccinate their children against influenza. We found that the intention to vaccinate is associated with influenza knowledge, trust in official information sources, responding parents’ education level, adherence to the NVP, number of children and change in childhood vaccination attitudes because of the COVID-19 pandemic. As expected, the choice of high and low vaccine coverage study areas translated to a statistically significant association between the areas and the intention to vaccinate: the intention to vaccinate was higher in the two known high coverage areas compared with the two low coverage areas. This underscores that the responders’ municipality of residence reflects existing regional features in vaccination intention.

In Finland, the low vaccine-coverage areas in the western parts of the country, where a high proportion of the population is Swedish-speaking, has been a topic of vaccine-related conversations [20]. In our study, language was not associated with the intention to vaccinate. Thus, mother tongue itself plays no significant role, but more likely reflects other differences between these areas. However, municipal-level analysis shows that adherence to the NVP was the only factor significantly associated with the intention to vaccinate in all study areas.

We found that the most reported reason for not vaccinating one’s child against influenza was the simple reason of the vaccine ‘not being offered’ at the clinic, which could be translated as ‘the nurse did not mention the vaccine’ or ‘I did not ask for the vaccine’. The result is concordant with the observation from the registry linkage that those with intention not to vaccinate maintained their standpoint (only 3% of children being vaccinated), but the vaccination was not realised for nearly one-fourth of children whose parents had vaccination intention. This underlines the need for public health professionals to proactively remind parents of the vaccination before and during the influenza season, in addition to offering easy access to the vaccine. Furthermore, there is a need for municipalities to promote influenza vaccination campaigns, which may boost awareness and prompt parents to proactively ask the public health nurse for the vaccine for their child. Both strategies are needed as not all children will have their routine check-up at the clinic during the influenza season, and hence the parents will not receive a reminder from the public health nurse. 

Our results are consistent with existing evidence of higher influenza-related knowledge being positively correlated with vaccination willingness [21]. We found a clear association between parental intention to vaccinate and influenza-related knowledge. However, only 74.6% of parents in our study were aware that children under the age of 7 years are entitled to a free seasonal influenza vaccine. In contrast, 93% of respondents knew that persons aged 65 years and above were entitled to an influenza vaccine. This finding poses the question whether the fact that parents simply do not know that their children are entitled to receive the vaccine plays a role in the suboptimal influenza vaccine uptake in the population of children in Finland.

In terms of vaccine-related information sources, only trusting official information was significantly associated with parental intention to vaccinate. This finding is in line with an observation that people living in Finland trust state institutions, scientific institutions and science in general [19]. The association between vaccine hesitancy and social media has been established in the literature [22]. However, in our study, trusting social media or influencers as vaccine-related information sources did not seem to affect parental decision to vaccinate one’s child against influenza.

It has been hypothesised that routine childhood vaccine hesitancy increased during the COVID-19 pandemic [23]. As parents receive vaccine-related information from many diverse sources, we examined whether parental intention to vaccinate their child against influenza was influenced by COVID-19 pandemic-induced vaccine hesitancy, caused by a potential change in parental risk perception. A recent Finnish study indicated that 66% of respondents felt that their trust towards science and scientists had increased during the pandemic [19]. Even though 14.6% of participants in our study indicated they have become more cautious, the majority (75.7%) indicated that their attitudes had remained the same.

The ability to verify the realisation of influenza vaccination of the participants’ children from the national vaccine register is a unique strength of the study. It enabled us to compare the parent’s initial vaccination intention and the realisation of said intention. After evaluating the reliability of parental answers in the survey by extracting the vaccination status for the child, it became evident that nearly all parents had already vaccinated their child against influenza before our data collection, i.e. before our study period February to March. According to the register, 50.8% of children of participating parents received their vaccination. Of these children, 96.2% were vaccinated before 1 February 2022. Hence, we can argue that our study did not substantially affect parental vaccine behaviour. This study is replicable in settings where childhood vaccinations are offered to children free of charge, and there is a chance to verify the actual uptake. It should also be noted that healthcare systems affect vaccination uptake, and healthcare systems vary across countries.

It is noteworthy that 50.8% of children whose parent took part in the study received the vaccination by the end of the 9-month follow-up period. This is lower than the initial vaccination intention for 64.0% of the children, but higher than the national coverage suggests. In contrast, most parents with no intention for the vaccination held firmly to their position, as only 3.0% of their children eventually received the vaccination. Parents who were unsure seemed to eventually accept the vaccine for their child, more so than parents who refused the vaccine, with 16.3% of their children eventually being vaccinated.

Our study has some limitations. Firstly, because of time constraints, our data collection started during the influenza season. An earlier data collection period would have provided us with a truer picture of intention and its realisation. However, by collecting the data during vs before the influenza season, we can exclude the potential effect that the differences in municipal-level influenza vaccine advertisements might have introduced. Secondly, despite receiving a reminder letter, the response rate for parents was only 13%, which is a major limitation. It is possible that parents found participation complicated because of the survey’s online format, which led to them to not participate. Furthermore, we cannot exclude potential bias from non-participation. As we collected answers from both parents, we recognise that this complicates the interpretation of the results. Moreover, after data collection, we had no means to differentiate between true intention (i.e. has not vaccinated but will) and realised intention (i.e. has vaccinated their child prior to participation) because of the wording of the survey question. However, the ability to examine the national vaccine register allowed us to verify the actual realisation of intention. Our study population may not be representative of the general population. Lastly, we did not ask parents about their child’s current state of health; pre-existing conditions could affect parental intention to vaccinate. 

## Conclusion

Our study provided insight into the understanding of factors associated with vaccination intentions. Parental influenza vaccination intention is associated with many factors and, in addition, even the positive intention does not always result in vaccination. To further explore the reasons affecting vaccination uptake, and to improve the communication between healthcare professionals and parents, a qualitative study including focus group discussions needs to be conducted.

## Supplementary Data

274000 Supplement
