# Supplementary material for: Factors associated with parental intention to vaccinate their child against influenza, Finland, February to March, 2022: a web-based survey
Source: Euro Surveill. 2023 Dec 7;28(49):2200828. doi: 10.2807/1560-7917.ES.2023.28.49.2200828 (PMC10831410; doi:10.2807/1560-7917.ES.2023.28.49.2200828)
Supplement: Supplement [file 22-00828_HUSSEIN_Supplement.pdf]

This supplementary material is hosted by *Eurosurveillance* as supporting information alongside the article Factors associated with parental intention to vaccinate their child against influenza, Finland, February to March, 2022: a web-based survey, on behalf of the authors, who remain responsible for the accuracy and appropriateness of the content. The same standards for ethics, copyright, attributions, and permissions as for the article apply. Supplements are not edited by *Eurosurveillance* and the journal is not responsible for the maintenance of any links or email addresses provided therein.

Sources of information and trust

1. How important do you think the following sources of information are in terms of vaccinations? Examples in brackets

	Very important	Rather important	Does not know	Not very important	Not at all important
Information produced by the authorities (Finnish Institute for Health and Welfare, Ministry of Social Affairs and Health, child health clinics, Wilma (=platform between schools and homes))					
Journalistic media (Helsingin Sanomat, Hufvudstadsbladet, tabloids, television)					
Social media (Facebook, Twitter, Youtube)					
Information produced by influencers) blogs, podcasts, profiles on social media)					
Personal contacts (family, circle of acquaintances, parents of coeval children)					
Other source, what? ___________________________					

2. How much do you trust vaccine information you receive from the following sources? Examples in brackets

	Very much	Rather much	Does not know	Not very much	Not at all
Information produced by the authorities (Finnish Institute for Health and Welfare, Ministry of Social Affairs and Health, child health clinics, Wilma)					
Journalistic media (Helsingin Sanomat, Hufvudstadsbladet, tabloids, television)					
Social media (Facebook, Twitter, Youtube)					
Information produced by influencers) blogs, podcasts, profiles on social media)					
Personal contacts (family, circle of acquaintances, parents of coeval children)					
Other source, what? ___________________________					

Expertise

3. How important do you find that a vaccine expert has the following qualities?

	Very important	Rather important	Do not know	Not very important	Not at all important
The expert represents a university or a research organisation					
The expert represents an authority					
The expert is actively in the public eye					
The expert has an appropriate scientific education					
The expert has practical experience on the matter					
The expert has good interaction skills					
The expert can communicate clearly					
When necessary, the expert has the ability to question the views of the authorities or scientists					
The expert represents the same sex as me					
I can relate to the expert					

4. Do any other qualities that an expert should have come to your mind?

____

Knowledge of influenza

5. Have you heard of influenza before?

- ☐ Yes
- ☐ No

6. Can influenza be treated with antibiotics?

- ☐ Yes
- ☐ No
- ☐ Does not know

7. For whom is the influenza vaccine recommended because of its significant benefits? Choose all that apply

- ☐ Children between the ages of 6 months and 6 years
- ☐ Pregnant women
- ☐ Social and healthcare, and pharmaceutical personnels
- ☐ Those over 65 years of age
- ☐ Those belonging to a risk group due to their illness or treatment
- ☐ Close circle of persons susceptible to severe influenza
- ☐ Men who start their conscription service and women who start their voluntary military service
- ☐ Does not know

8. Can the influenza vaccine cause influenza?

- ☐ Yes
- ☐ No
- ☐ Does not know

9. Can a person infected with the flu spread the disease before symptoms start?

- ☐ Yes
- ☐ No
- ☐ Does not know

10. How would you rate your knowledge on influenza and its preventive vaccination?

- ☐ Very good
- ☐ Good
- ☐ Moderate
- ☐ Weak
- ☐ Very weak
- ☐ Do not know

Vaccination behaviour

11. Has your child received the (childhood) vaccines included in the national vaccination programme?

- ☐ Yes, all offered vaccines so far (jump to question 12)
- ☐ Yes, some of the offered vaccines so far (jump to question 11b)
- ☐ Not a single offered vaccine (jump to question 11b)
- ☐ Do not know (jump to question 12)

11b. If you answered ”yes, some of the offered vaccine” or “not a single offered vaccine”, what was the reason for not vaccinating? Choose all that apply

- ☐ I wanted to wait until my child was a bit older
- ☐ I did not believe the vaccine is efficient/useful
- ☐ The side effects of the vaccine worried me
- ☐ The vaccine was not given due to the COVID-19 pandemic
- ☐ There is a person who has suffered serious harm from the vaccine in my inner circle
- ☐ My child is afraid of needles
- ☐ I had read or heard negative things about the vaccine
- ☐ I did not have comprehensive and reliable information of the vaccine
- ☐ The vaccine has not been studied enough in my opinion
- ☐ I was overall suspicious of the vaccine
- ☐ The appointment offered to my child did not fit my schedule
- ☐ My child refused
- ☐ Other reason, what? _____________________________________

12. With whom do you mainly consider vaccine-related issues? Choose all that apply

- ☐ Healthcare professionals (doctor, public health nurse, child health clinic etc.)
- ☐ The child’s other parent
- ☐ Family other than the child’s other parent
- ☐ Circle of acquaintances
- ☐ Parents of other coeval children
- ☐ Social media contacts
- ☐ Other, who? _______________________________________

13. Who ultimately decides on the vaccination of your child?

- ☐ Me
- ☐ The child’s other parent
- ☐ We decide jointly with the child’s other parent
- ☐ The child themself
- ☐ Family other than the child’s other parent
- ☐ Other, who? ______________________________

14. My child has or will receive the influenza vaccine free of charge as per the national vaccination programme

- ☐ Yes, has received or will receive (jump to question 14a)
- ☐ No, has not received or will not receive (jump to question 14b and 14c)
- ☐ Do not know (jump to question 14d)

14a. How much influence the following entities have had on your decision to vaccinate your child against influenza?

	No influence at all	Little influence	Somewhat of an influence	Very much influence	Do not know
Healthcare professionals (doctor, public health nurse, child health clinic etc.)					
The child’s other parent					
Family other than the child’s other parent					
Circle of acquaintances					
Parents of other coeval children					
Media, press					
Social media contacts					
Social media influencers					
Local influencer, who?					
Other, what/who?					

14b. What is/are the reasons that your child **has not** or **will not** receive the influenza vaccine this season? Choose all that apply

- ☐ I do not think the disease is dangerous
- ☐ I believe that the risk to acquire the condition is small
- ☐ I want to wait until the child is a bit older
- ☐ I did not think the vaccine was efficient/useful
- ☐ The adverse effects of the vaccine worry me
- ☐ There is a person in my vicinity who has suffered adverse effects from the vaccine
- ☐ My child is afraid of needles
- ☐ I had read or heard negative things about the vaccine
- ☐ I needed comprehensive and reliable information of the vaccine
- ☐ The vaccine has not been studied enough in my opinion
- ☐ I was overall suspicious of the vaccine
- ☐ The appointment offered to my daughter did not fit my schedule
  - (if chosen, more questions appear) What describes your situation the best:
    - The public health nurse did not mention the vaccine at all
    - The public health nurse mentioned it, but did not give an opinion
    - The public health nurse told me that my child should not take the vaccine
    - Other reason
- ☐ I do not trust health authorities (Ministry for Social Affairs and Health, Finnish Institute for Health and Welfare, Fimea (Finnish Medical Agency))
- ☐ I do not trust the professional skills of the healthcare professionals

☐ Other, what?:____

14c. How much influence do the following entities have had on your decision to not vaccinate your child against influenza?

	No influence at all	Little influence	Somewhat of an influence	Very much influence	Do not know
Healthcare professionals (doctor, public health nurse, child health clinic etc.)					
The child's other parent					
Family other than the child's other parent					
Circle of acquaintances					
Parents of other coeval children					
Media, press					
Social media contacts					
Social media influencers					
Local influencer, who?					
Other, what/who?					

14d. How much influence do the following entities have had on your decision to vaccinate?

	No influence at all	Little influence	Somewhat of an influence	Very much influence	Do not know
Healthcare professionals (doctor, public health nurse, child health clinic etc.)					
The child's other parent					
Family other than the child's other parent					
Circle of acquaintances					
Parents of other coeval children					
Media, press					
Social media contacts					
Social media influencers					
Local influencer, who?					
Other, what/who?					

15. What is your biggest concern relating to influenza and its vaccine?

____

16. My attitude regarding children's vaccines has changed along the COVID-19 pandemic and its' vaccine

☐ Yes, my attitude has become more trusting towards children's vaccines
☐ Yes, my attitude has become more cautious towards children's vaccines
☐ No, my attitude has not changed towards children's vaccines

Background information

17. Year of birth: ____

18. Child’s birth month and year: ___/20___

19. How many children are there in your family currently? __________

20. Do you smoke?

- ☐ Yes
- ☐ No
- ☐ Not anymore

21. Parent’s relationship

- ☐ Marriage or cohabitation, only joint children
- ☐ Marriage or cohabitation, children from different parents
- ☐ Lone parent
- ☐ Lone parent, children from different parents
- ☐ Other

22. Mother tongue:

- ☐ Finnish
- ☐ Swedish
- ☐ Other, what? ____________________

23. The educational level of the responding parent (choose highest completed)

- ☐ Middle or primary school
- ☐ Vocational school
- ☐ Upper secondary school
- ☐ Lower higher education degree (e.g., bachelor’s degrees)
- ☐ Higher education degree (e.g., master’s degrees)
- ☐ Doctoral degree (e.g., licentiate, PhD)
- ☐ No information

24. The educational level of the child’s other parent (choose highest completed)

- ☐ Middle or primary school
- ☐ Vocational school
- ☐ Upper secondary school
- ☐ Lower higher education degree (e.g., bachelor’s degrees)
- ☐ Higher education degree (e.g., master’s degrees)
- ☐ Doctoral degree (e.g., licentiate, PhD)
- ☐ No information

25. What occupational group would you categorise yourself to?

- ☐ Leading position
- ☐ Clerk
- ☐ Employee
- ☐ Entrepreneur/freelancer
- ☐ Farmer
- ☐ Student
- ☐ Stay at home parent
- ☐ Unemployed
- ☐ Other, what?

26. Which religious group would you categorise yourself to? Choose all that apply

- ☐ Evangelical Lutheran or Eastern Orthodox church
    - ☐ Some revivalist movement within the church

- ☐ Other Christian religion
- ☐ Other, what? ________________________
- ☐ No religion
- ☐ Do not want to say

27. The combined gross income of the family (salary, parental allowances, unemployment benefit etc.)

- ☐ <10 000 Euros annually
- ☐ 10–20 000
- ☐ 20–40 000
- ☐ 40–60 000
- ☐ 60–80 000
- ☐ 80–100 000
- ☐ >100 000
- ☐ Do not want to say

28. After this questionnaire study we will organise focus group discussions. The aim for the discussions is to further explore parental media use in terms of vaccines and trust. Would you be interested in partaking focus group discussion organized in your municipality?

- ☐ Yes
- ☐ No
